# Rhythm versus rate control in patients with newly diagnosed atrial fibrillation – Observations from the GARFIELD-AF registry

**DOI:** 10.1016/j.ijcha.2023.101302

**Published:** 2023-11-16

**Authors:** Marita Knudsen Pope, Trygve S. Hall, Saverio Virdone, Dan Atar, A. John Camm, Karen S Pieper, Petr Jansky, Sylvia Haas, Shinya Goto, Elizaveta Panchenko, Gonzalo Baron-Esquivias, Pantep Angchaisuksiri, Ajay K Kakkar, for the GARFIELD-AF Investigators

**Affiliations:** a Institute of Clinical Medicine, University of Oslo, Oslo, Norway; b Department of Cardiology, Oslo University Hospital, Ullevål, Oslo, Norway; c Thrombosis Research Institute, London, the United Kingdom of Great Britain and Northern Ireland; d Cardiology Clinical Academic Group Molecular & Clinical Sciences Research Institute, St. George’s University of London, London, the United Kingdom of Great Britain and Northern Ireland; e Department of Cardiovascular Surgery, Motol University Hospital, Prague, Czech Republic; f Sylvia Haas: Formerly Department of Medicine, Technical University of Munich, Munich, Germany; g Tokai University, Kanagawa, Japan; h National Medical Research Center of Cardiology of Ministry of Health of the Russian Federation, Moscow, Russian Federation; i Servicio de Cardiología y Cirugía Cardíaca, Hospital Universitario Virgen del Rocío., Universidad de Sevilla., Sevilla. Departamento Cardiovascular, Instituto de Biotecnología de Sevilla (IBIS), Spain; j Department of Medicine, Ramathibodi Hospital, Mahidol University, Thailand

**Keywords:** Atrial fibrillation, Rhythm control, Rate control, Stroke, Mortality, Real-world evidence

## Abstract

**Background:**

Investigate real-world outcomes of early rhythm versus rate control in patients with recent onset atrial fibrillation.

**Methods:**

The Global Anticoagulant Registry in the FIELD-AF (GARFIELD-AF) is an international multi-centre, non-interventional prospective registry of newly diagnosed (≤6 weeks’ duration) atrial fibrillation patients at risk for stroke. Patients were stratified according to treatment initiated at baseline (≤48 days post enrolment), and outcome risks evaluated by overlap propensity weighted Cox proportional-hazards models.

**Results:**

Of 45,382 non-permanent atrial fibrillation patients, 23,858 (52.6 %) received rhythm control and 21,524 (47.4 %) rate control. Rhythm-controlled patients had lower median age (68.0 [Q1;Q3: 60.0;76.0] versus 73.0 [65.0;79.0]), fewer histories of stroke/transient ischemic attack/systemic embolism (9.4 % versus 13.0 %), and lower expected probabilities of death (median GARFIELD-AF death score 4.0 [2.3;7.5] versus 5.1 [2.8;9.2]). The two groups had the same median CHA_2_DS_2_-VASc scores (3.0 [2.0;4.0]) and similar proportions of anticoagulated patients (rhythm control: 66.0 %, rate control: 65.5 %). The propensity-score-weighted hazard ratios of rhythm vs rate control (reference) were 0.85 (95 % CI: 0.79–0.92, p-value < 0.0001) for all-cause mortality, 0.84 (0.72–0.97, p-value 0.020) for non-haemorrhagic stroke/systemic embolism and 0.90 (0.78–1.04, p-value 0.164) for major bleeding.

**Conclusion:**

Rhythm control strategy was initiated in about half of the patients with newly diagnosed non-valvular non-permanent atrial fibrillation. After balancing confounders, significantly lower risks of all-cause mortality and non-haemorrhagic stroke were observed in patients who received early rhythm control.

## Graphical abstract

**Figure:**
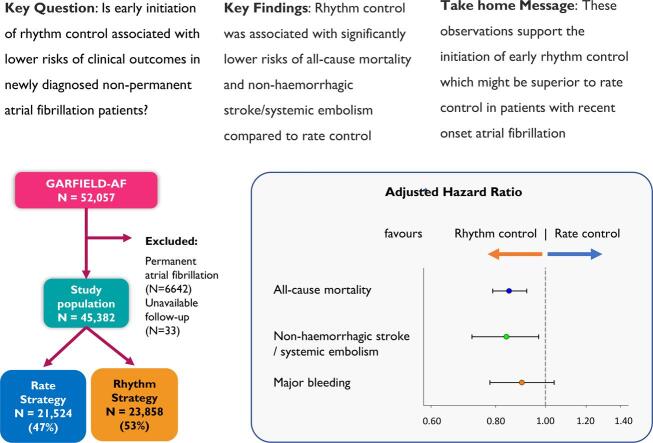

## 1 Introduction

Atrial fibrillation (AF) is associated with major health challenges, such as an increased risk of stroke [1], [2], heart failure [3], [4], hospitalisation [5], [6], cognitive impairment and dementia [7], [8], and mortality [1], [9], [10]. The condition itself also causes life-altering symptoms which can result in reduced quality-of-life [11]. Public health care costs related to AF are significant [6], [12], [13] and AF represents a growing health burden. Therefore, optimal management of these patients represents important health economic and societal issues.

Over the last years, evidence in support of the beneficial effects of early rhythm control has been increasing [14]. Treatments that achieve and maintain sinus rhythm can reduce symptom burden [15], prevent left atrial remodelling [16] and delay the progression of AF [17], [18], especially if initiated at an early stage. However, randomized controlled trials (RCTs) comparing rates of cardiovascular outcomes for rhythm vs rate control strategies produced conflicting results [19], [20], [21], [22], [23]. Moreover, real-world data on the outcomes of early rhythm or rate control in patients with newly diagnosed AF are limited to specific recruitment sites, patient populations, treatment methods, or AF history of up to one year.

We explored early rhythm control versus rate control strategies and their clinical outcomes in the Global Anticoagulant Registry in the FIELD-AF (GARFIELD-AF), a non-interventional registry of real-world adult patients with newly diagnosed AF.

## 2 Methods

### 2.1 Study design

This is an international, prospective, observational study using registry data from GARFIELD-AF [24]. Patients were diagnosed with AF within six weeks - on average two weeks - before enrolment, and had at least one risk factor for stroke. Risk of stroke was determined by the local investigator, and was not restricted to a specific risk factor or risk scoring scheme. Patients with valvular disease or a transient cause of AF were not included in the registry. For the purpose of this study, patients with permanent AF were also excluded. Enrolment took place in 1317 sites in 35 countries. The sites were randomly selected, except for 18 sites where the national lead investigators had to recommend additional sites to reach the required number in the given country. Study sites represent different care settings in each country. Patients were enrolled in five consecutive cohorts in the period from March 2010 to August 2016. Follow-up time was two years from enrolment.

### 2.2 Procedures

Clinical characteristics, including medical history, care setting, type of AF, and treatment choice, were documented at inclusion. Follow-up data were collected at 4-month intervals for up to two years post enrolment. Outcomes were investigator reported. Data were collected in electronic case report forms designed by Dendrite Clinical Systems Ltd (Henley-on-Thames, UK). Submitted information was examined for accuracy and completeness by the coordinating centre, the Thrombosis Research Institute (London, UK). Quality control included both on-site audits and remote measures. Twenty percent of all electronically reported data were automatically monitored against source documentation [25]. Data for the present analysis were extracted from the final locked registry database in June 2019.

### 2.3 Definitions, variables, and outcome measures

Stratification to rhythm or rate control was based on treatment strategy initiated at baseline, defined as within 48 days of enrolment. This time-window was based on mandatory effective anticoagulation therapy for patients prior to rhythm control. Rhythm control was defined as investigator reported initiation of a rhythm control strategy, including treatment with antiarrhythmic drug(s), cardioversion, or ablation – alone or in combination with rate modifiers. Antiarrhythmic drugs included class 1a, class 1c and class III antiarrhythmics. A recent *meta*-analysis found no differences in the rates of all-cause mortality, thromboembolic events, or myocardial infarction between Class I and/or III antiarrhythmics versus control [26]. Beta-blockers and Digoxin were categorised as rate modifiers. Rate control was defined as investigator reported initiation of a rate control strategy and absence of rhythm control therapy.

Vascular disease included patients with coronary artery disease and/or peripheral artery disease. Chronic kidney disease was classified into moderate-to-severe (stages 3–5), mild (stages 1 and 2), or none, according to National Kidney Foundation guidelines (https://kdigo.org/guidelines/ckd-evaluation-and-management/). Heart failure (HF) was defined as prior or current HF or left ventricular ejection fraction < 40 %. AF type was classified according to the European Society of Cardiology guidelines [15]. Several scores were used for assessment of risk: the GARFIELD-AF risk calculator (representing expected occurrence of mortality, transient ischaemic attack (TIA)/systemic embolism (SE) or major bleeding within two years from baseline) [27], the CHA_2_DS_2_-VASc score (HF, hypertension, age ≥ 75 years, diabetes, ischaemic stroke/TIA/SE, vascular disease, age 65–74 years, female sex) [28], and a modified HAS-BLED score (systolic blood pressure > 160 mmHg, abnormal renal function, abnormal liver function, stroke history, bleeding history, age > 65 years, use of platelet inhibitors or non-steroidal anti-inflammatory drugs, > 8 units of alcohol per week, but not labile INR).

Outcome measures were non-haemorrhagic stroke or SE, major bleeding, all-cause, cardiovascular and non-cardiovascular mortality, and new or worsening HF. Non-haemorrhagic stroke/SE was a composite of ischaemic or unknown-type stroke and SE. Major bleeding was reported by investigators according to the International Society on Thrombosis and Haemostasis (ISTH) definition [29]. Minor/non-major clinically relevant bleeds that occurred in a critical site or required transfusion were reclassified as major bleeding. Worsening HF was defined as re-stratification into higher New York Heart Association (NYHA) classification after enrolment or acute or progressive decompensation of previous stable HF.

### 2.4 Statistical analysis

Baseline characteristics are expressed as median (interquartile range) for continuous variables, and frequency and percentage for categorical variables. Clinical outcomes are reported as number of events and event rate per 100 person-years with 95 % confidence interval (CI). A Poisson model was used to estimate person-year rates. The follow-up period was from the date of enrolment, truncated at first event occurrence (with regard to the outcome of interest), death, loss to follow-up, or two years after enrolment, whichever occurred first.

A Cox proportional hazards model, using a propensity method of overlap weighting, was used to assess the effect of rhythm versus rate control on clinical endpoints [30]. In brief, this applied method overlaps weights and optimizes the efficiency of comparisons by defining the population with the most overlap in the covariates between treatment groups. This scheme eliminates the potential for outlier weights by avoiding a weight based on a ratio calculation, using values bounded by 0 and 1. Thus, when using overlap weights, many of the concerns regarding the assessment and trimming of weights are eliminated. Balance of covariates before and after the application of the weighting scheme, quantified through absolute standardised differences, and the propensity score distribution in the rate, rhythm and overlap groups are reported in the supplementary material (Figures S1 and S2). Covariates evaluated in the weighting scheme included demographic characteristics, medical history, and other characteristics (Table S1).

Only complete cases were presented in descriptive tables. Multiple imputation was applied in the comparative effectiveness analyses to examine the effect of rhythm control on outcomes [31]. Standard errors were obtained by combining estimates across five imputed datasets. Two sensitivity analyses were performed, excluding either patients with new onset (unclassified) AF, or patients recruited in primary care. We performed four subgroup analyses, exploring outcomes for patients with and without heart failure, with and without AF-related symptoms, with CHA2DS2-VASc > 4 and with ≤ 4, and patients initiated on rhythm control within two days of diagnosis and those initiated on rhythm control between three and 42 days of diagnosis. For the analysis by symptoms at baseline, patients were considered symptomatic if they had at least one of the following clinical features documented at baseline: palpitations, shortness of breath, chest pain/discomfort, dizziness, tiredness, sweating, or fainting, and those with signs such as irregular pulse or tachycardia but no symptoms were considered asymptomatic. Statistical analyses were carried out using SAS (version 9.4).

### 2.5 Ethics

The GARFIELD-AF study protocol was approved by independent ethics committees and/or hospital-based institutional review boards. The study is in accordance with the principles of the Declaration of Helsinki and the International Conference on Harmonisation – Good Pharmaco-epidemiological and Clinical Practice guidelines. All participating patients signed an informed consent form prior to enrolment.

## 3 Results

A total of 52 057 patients were enrolled in the GARFIELD-AF registry from 2010 to 2016. For the current analysis, patients with permanent type of AF (n = 6 642) and/or missing follow-up information (n = 33) were excluded. Of the 45 382 assessed patients, 23 858 (52.6 %) received rhythm control and 21 524 (47.4 %) rate control.

**Fig. 1:**
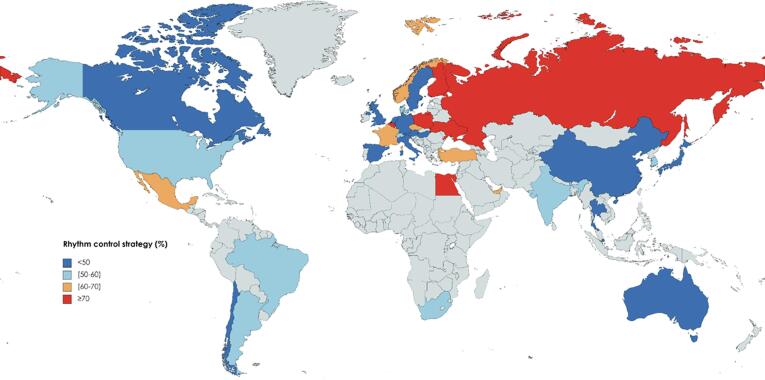
Distribution of rhythm control strategy initiated at diagnosis by country of enrolment.

Rates of rhythm control were similar throughout the study period (52.7 % in 2010/2011, 54.2 % in 2015/2016) (Figure S3). Distribution of treatment strategies varied by geographic regions (Fig. 1, Table S2). The majority of patients were diagnosed in hospital (71.2 %). Patients diagnosed by cardiologists were more likely to receive rhythm control (57.3 %) than patients diagnosed by internists, geriatricians or neurologists (43.92 %) or general practitioners (41.1 %). Females made up 44.3 % of the study and among the female patients, 52.1 % received rhythm control.

### 3.1 Baseline characteristics

**Fig. 2:**
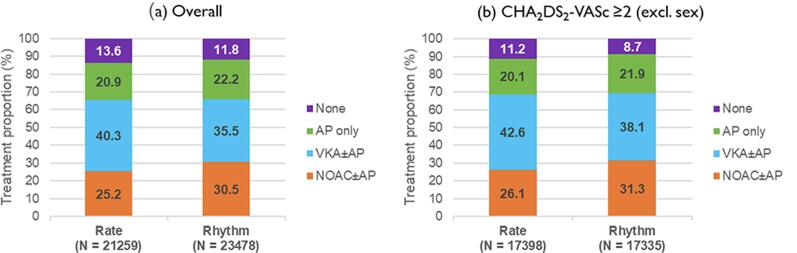
Distribution of baseline antithrombotic treatment^1^ by treatment strategy initiated at baseline a) overall and b) among patients with CHA_2_DS_2_-VASc ≥ 2, excl. sex). ^1^ Patients with unavailable antithrombotic treatment information are excluded from this analysis (N = 645 overall and N = 523 among patients with CHA_2_DS_2_-VASc ≥ 2, excl. sex).

Patients in the rhythm control group were younger (68.0 (60.0;76.0) versus 73.0 (65.0;79.0) years), had lower rates of prior stroke/TIA/SE (9.4 % versus 13.0 %) and moderate to severe chronic kidney disease (8.7 % versus 12.3 %), and a lower median GARFIELD-AF death score (4.0 (2.3;7.5) versus 5.1 (2.8;9.2)) (Table 1). Fewer patients in the rate control group had paroxysmal AF. Median CHA_2_DS_2_-VASc scores were 3.0 (2.0;4.0) in both groups. Rate of anticoagulation treatment was similar in the rhythm and rate control group, both in the overall study population (66.0 % versus 65.5 %) and in patients with CHA_2_DS_2_-VASc score ≥ 2 (excl. sex) (69.4 % versus 68.7 %) (Fig. 2). The use of ACEi/ARB, MRA, beta-blockers, antihypertensive drugs, and statins were similar in both treatment arms (table S3). The distribution of treatment methods (antiarrhythmic medication, cardioversion, or ablation) is shown in table S4. Patients in the rhythm control group were more frequently symptomatic than those in the rate control group (80.9 % vs 69.9 % with at least one symptom). Distribution of reported symptoms can be found in the supplementary material (table S5).

**Table 1:** Baseline characteristics by treatment strategy initiated at diagnosis.

Baseline characteristics	Treatment strategy	
	Rate control (N = 21524)	Rhythm control (N = 23858)
Sex, n (%)		
Male	11,878 (55.2)	13,379 (56.1)
Female	9646 (44.8)	10,479 (43.9)

Age, median (Q1; Q3), years	73.0 (65.0;79.0)	68.0 (60.0;76.0)

Ethnicity, n (%)		
Caucasian	12,221 (58.4)	15,145 (65.0)
Hispanic/Latino	1241 (5.9)	1524 (6.5)
Asian	7077 (33.8)	6071 (26.1)
Afro-Caribbean/Mixed/Other	377 (1.8)	553 (2.4)

BMI, median (Q1; Q3), kg/m^2^	26.5 (23.6;30.4)	27.1 (24.2;30.9)
Systolic blood pressure, median (Q1; Q3), mmHg	131.0 (120.0;145.0)	130.0 (120.0;144.0)
Diastolic blood pressure, median (Q1; Q3), mmHg	80.0 (70.0;88.0)	80.0 (70.0;89.0)
Pulse, median (Q1; Q3), bpm	84.0 (71.0;102.0)	84.0 (70.0;110.0)

Type of atrial fibrillation, n (%)		
Persistent	4001 (18.6)	3752 (15.7)
Paroxysmal	5512 (25.6)	8792 (36.9)
New onset (unclassified)	12,011 (55.8)	11,314 (47.4)

Care setting specialty at diagnosis, n (%)		
Internal medicine/Neurology/Geriatrics	5041 (23.4)	3948 (16.5)
Cardiology	13,018 (60.5)	17,461 (73.2)
Primary care/General practice	3464 (16.1)	2449 (10.3)

Care setting location at diagnosis, n (%)		
Hospital	12,327 (57.3)	14,647 (61.4)
Office/Anticoagulation clinic/thrombosis centre	6870 (31.9)	6182 (25.9)
Emergency room	2326 (10.8)	3029 (12.7)

Medical history, n (%)		
Heart failure	4539 (21.1)	5365 (22.5)
Acute coronary syndromes	2157 (10.1)	2714 (11.4)
Vascular disease^1^	4931 (23.1)	6177 (26.0)
Carotid occlusive disease	653 (3.1)	646 (2.7)
VTE	603 (2.8)	562 (2.4)
Prior stroke/TIA/SE	2769 (13.0)	2216 (9.4)
Prior bleeding	608 (2.8)	519 (2.2)
Hypertension	16,328 (76.1)	18,118 (76.1)
Hypercholesterolaemia	8423 (40.5)	9888 (42.8)
Diabetes	4928 (22.9)	5102 (21.4)
Cirrhosis	155 (0.7)	107 (0.5)
Moderate to severe CKD	2523 (12.3)	2006 (8.7)
Dementia	388 (1.8)	236 (1.0)

Heavy alcohol consumption, n (%)	477 (2.7)	452 (2.2)
Current smoker, n (%)	2018 (10.4)	2660 (12.2)

Treatment, n (%)		
NOAC ± AP	5352 (25.2)	7160 (30.5)
VKA ± AP	8564 (40.3)	8333 (35.5)
AP only	4443 (20.9)	5214 (22.2)
None	2900 (13.6)	2771 (11.8)
AP ± OAC, n (%)	7262 (34.2)	8726 (37.2)

CHA2DS2-VASc score, median (Q1; Q3)	3.0 (2.0;4.0)	3.0 (2.0;4.0)
CHA_2_DS_2_-VASc score, mean (SD)	3.4 (1.6)	3.0 (1.6)
HAS-BLED score, median (Q1; Q3)^2^	1.0 (1.0;2.0)	1.0 (1.0;2.0)
HAS-BLED score, mean (SD)^2^	1.5 (0.9)	1.3 (0.9)
GARFIELD-AF death score, median (Q1; Q3)^3^	5.1 (2.8;9.2)	4.0 (2.3;7.5)
GARFIELD-AF death score, mean (SD)^3^	7.6 (7.9)	6.1 (6.4)
GARFIELD-AF stroke score, median (Q1; Q3)^4^	1.7 (1.2;2.6)	1.4 (1.0;2.1)
GARFIELD-AF stroke score, mean (SD)^4^	2.1 (1.6)	1.8 (1.3)
GARFIELD-AF bleeding score, median (Q1; Q3)^5^	1.7 (1.1;2.6)	1.4 (0.9;2.2)
GARFIELD-AF bleeding score, mean (SD)^5^	2.1 (1.5)	1.7 (1.3)

BMI: body mass index, VTE: venous thromboembolism, TIA: transient ischemic attack, SE: systemic embolism, CKD: chronic kidney disease, OAC: oral anticoagulant, VKA: vitamin K antagonist, NOAC: non-VKA OAC, AP: anti-platelet treatment.

1 Defined as peripheral artery disease and/or coronary artery disease.

2 The risk factor ‘Labile INRs’ is not included in the HAS-BLED score as it is not collected at baseline. As a result, the maximum HAS-BLED score at baseline is 8 points (not 9).

3 Represents the expected risk of mortality within two years.

4 Represents the expected risk of non-haemorrhagic stroke within two years.

5 Represents the expected risk of major bleeding within two years.

### 3.2 Clinical outcomes

Event rates per 100 person-years over two years follow-up in the rhythm and rate control group were 2.94 (95 % CI: 2.78–3.10) and 4.43 (4.22–4.64) for mortality, 0.84 (0.75–0.92) and 1.16 (1.05–1.27) for non-haemorrhagic stroke/SE and 0.84 (0.76–0.93) and 1.16 (1.06–1.27) for major bleeding (Table S6).

**Fig. 3:**
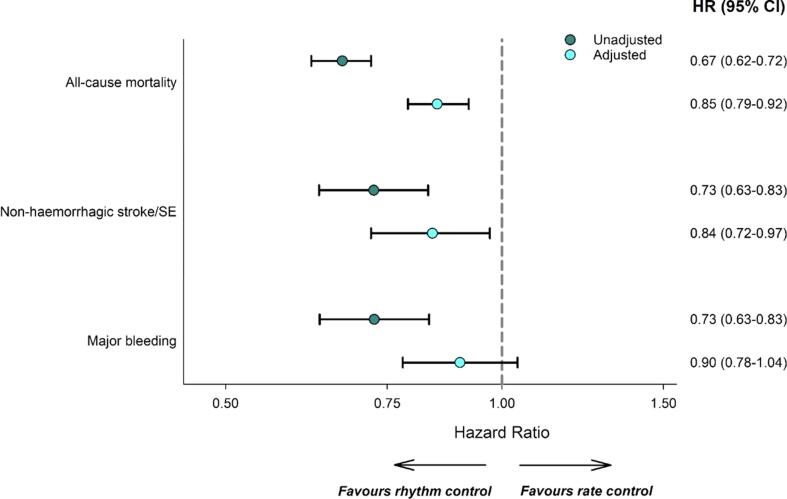
Unadjusted and adjusted hazard ratios^1^ for selected outcomes within two-year follow-up by treatment strategy initiated at diagnosis. ^1^Obtained using an overlap-weighted Cox model. Variables included in the weighting scheme are: country and cohort enrolment, sex, age, ethnicity, type of atrial fibrillation (AF), care setting speciality and location, congestive heart failure, acute coronary syndromes, vascular disease, carotid occlusive disease, prior stroke/transient ischemic attack (TIA)/ systemic embolism (SE), prior bleeding, venous thromboembolism (VTE), hypertension, hypercholesterolemia, diabetes, cirrhosis, moderate to severe chronic kidney disease (CKD), dementia, hyperthyroidism, hypothyroidism, current smoking, heavy alcohol consumption, body mass index (BMI), heart rate, systolic and diastolic blood pressure at diagnosis, antiplatelet and anticoagulant initiated at baseline.

After propensity score overlap weighting, patients of the two groups were balanced for all observed characteristics (Figure S1). Adjusted hazard ratios (95 % CI) within two years follow-up were 0.85 (0.79–0.92, p-value < 0.0001) for all-cause mortality, 0.75 (0.69–0.89, p-value < 0.001) for cardiovascular mortality, 0.94 (0.83–1.07, p-value 0.34) for non-cardiovascular mortality, 0.84 (0.72–0.97, p-value 0.02) for non-haemorrhagic stroke/SE and 0.90 (0.78–1.04, p-value 0.16) for major bleeding (Fig. 3). Cumulative incidence curves for the outcomes can be found in Figure S4.

In the two sensitivity analyses performed, the results were similar to the main analysis, with the exception that no significant difference in non-haemorrhagic stroke/SE was found in the analysis excluding patients with new onset (unclassified) AF (table S7) or patients diagnosed in primary care (table S8).

Tables S9 and S10 depict details on subgroup analyses of patients with and without HF. In patients with HF, all-cause mortality was significantly lower in the rhythm control group. For patients without HF the result pointed in the same direction, although the outcome was non-significant with a p-value of 0.053. Cardiovascular mortality was, however, significantly lower (p-value < 0.001). Patients without HF had a lower risk of non-haemorrhagic stroke/SE in the rhythm control group, while the confidence interval for this outcome was wide with a high degree of uncertainty in patients with HF. The majority of patients (74 %) had at least one symptom at diagnosis, and the results of the subgroup analysis of patients with symptoms reflect the ones seen in the overall group (table S9). In patients without symptoms, the associations seem attenuated with no statistical significance for the endpoints (table S10).

To examine a possible interaction with risk of stroke, rhythm vs rate propensity score weighted analyses was performed in patients with CHA2DS2-VASc > 4 and with ≤ 4. The beneficial estimates for all-cause and cardiovascular mortality were very similar in high and lower risk patients (tables S11 and S12).

Subgroup analyses of patients initiated on rate or rhythm control at diagnosis, stratified by those who were enrolled within two days of AF diagnosis and those enrolled between three and 42 days of diagnosis, were also performed. Small differences in outcomes were found compared to the main analysis (tables S13 and S14).

## 4 Discussion

In this large, prospective, observational study of patients with newly diagnosed AF, early initiation of a rhythm control strategy was associated with lower adjusted risks of all-cause mortality and non-haemorrhagic stroke.

A rhythm control strategy was initiated early after diagnosis in about half of the patients, similar to what has been reported from other AF registries from the same time [32], [33], [34]. The proportion of patients receiving rhythm control remained stable throughout the period of enrolment. This might be expected because guidelines for rhythm control did not change during these years [35], [36], [37].

Patients diagnosed by cardiologists were more likely to receive rhythm control than patients diagnosed by internists, geriatricians, neurologists or general practitioners. The same trend was seen in an observational study from Quebec [38]. Factors contributing to differences in treatment choice among medical specialities might include different patient samples, patients assigned for cardioversion being referred to a cardiologist (and thus recruited to the study in this setting), or cardiologists being more positive about rhythm control. Choice of treatment strategy varied from country to country, and additional therapy, patient lifestyle and follow-up resources might have differed between the regions. These factors may possibly have contributed to the differences observed between the rhythm and rate control group.

Patients in the rhythm control group were younger and had a lower risk profile at baseline, with lower GARFIELD-AF risk scores for death and stroke. The median CHA₂DS₂-VASc score and proportion of patients on anticoagulation treatment was the same in both groups. Patients in the rhythm control group had a higher percentage of paroxysmal AF. Given the natural history AF, they were likely to be in earlier stage of the disease which might have facilitated the decision to deploy rhythm control. After adjustment for baseline risk factors, rhythm control was associated with significantly lower rates of mortality and non-haemorrhagic stroke over two years of follow-up. In the Early Treatment of Atrial Fibrillation for Stroke Prevention (EAST-AFNET 4) trial, early rhythm control reduced cardiovascular death, stroke, and hospitalization for heart failure and acute coronary syndrome regardless of AF pattern. Moreover, early rhythm control improved health-related quality of life in patients with paroxysmal and persistent, but not with first diagnosed AF [39].

Several previous RCTs, including AFFIRM (Atrial Fibrillation Follow-up Investigation of Rhythm Management) [21], AF-CHF (Atrial Fibrillation and Congestive Heart Failure) [20], RACE (Rate Control Versus Electrical Cardioversion for Persistent Atrial Fibrillation Study) [22], have shown that rhythm control is non-superior to rate control when considering hard endpoints. In these studies, however, patients often had long-standing AF. In the ATHENA study (A Placebo-Controlled, Double-Blind, Parallel Arm Trial to Assess the Efficacy of Dronedarone 400 mg bid for the Prevention of Cardiovascular Hospitalization or Death from Any Cause in Patients with Atrial Fibrillation/Atrial Flutter) [40], patients with a short time in AF who received dronedarone had a lower risk of cardiovascular hospitalisation and cardiovascular death, and a post hoc analysis also found that the population with recent onset AF had a lower risk of these events [41]. A second post hoc analysis from this study demonstrated a lower risk of stroke [42]. Furthermore, in the EAST-AFNET 4 trial that included patients diagnosed within a year before enrolment, a rhythm control strategy was associated with lower risk of cardiovascular outcomes [43], including individual endpoints of cardiovascular death and stroke. In addition to being initiated on treatment early after diagnosis, some of the differences observed in the EAST-AFNET 4 study may be explained by more use of ablation and contemporary rhythm control medication with a better risk profile than the medication given to patients in the older RCTs mentioned above. This is also supported by a post hoc analysis from the AFFIRM study, which found no significant difference in outcomes between rhythm and rate control strategies in patients diagnosed with AF within six months of enrolment [44].

However, early rhythm control was associated with better outcomes in several registry studies [45], [46], [47]. A nationwide database study in Korea found that patients on early rhythm control (initiated within one year of diagnosis) had a lower risk of adverse cardiovascular events, defined as a composite outcome of death from cardiovascular causes, ischemic stroke, admission to hospital for heart failure, or acute myocardial infarction [46]. Compatible results were observed in a large United States health care database study [47]. Furthermore, a recently published GARFIELD-AF sub-group analysis showed that patients with newly diagnosed AF had a significantly lower rate of mortality when receiving early cardioversion compared to no cardioversion therapy [48]. The current work is expanding on these results by integrating all forms of rhythm control, including but not limited to cardioversion. Our observations support the findings of the earlier studies. Early initiation of a rhythm control strategy might be superior to rate control in patients with recent onset AF.

### 4.1 Strengths and limitations

As mentioned above, published studies from national health databases [45], [46], [47] and different cohort studies [32], [33], [34], [49], [50] have addressed different sides of rhythm vs rate control in patients with recently diagnosed AF. Information gathered routinely in general health records may differ in content from what is gathered in specific patient registries. In the GARFIELD-AF registry, a wide range of relevant information for patients with recently diagnosed AF was collected in a systematic, uniform way. To ensure high data quality, a comprehensive audit and quality control system was enacted**.** The current study was prospective, unlike the retrospective analyses of national registries, lowering the risk of potential bias [51]. In contrast to the cohort studies mentioned above, patients in the GARFIELD-AF registry had very recent AF, diagnosed on average two weeks before enrolment.

RCTs are the gold standard for examining cause-effect relationships, but use strict patient enrolment criteria and treatment instructions. Conversely, patients included in observational studies like ours might have risk profiles and clinical outcomes that better represent those in everyday clinical practice. GARFIELD-AF patients were enrolled both in hospital and outpatient clinics, by primary care physicians and cardiologists and other specialty physicians from representative study sites world-wide. Patients were not excluded based on estimated survival, presence of other comorbidities or treatments, and the rhythm control group included all rhythm control treatment options. Hence, the GARFIELD-AF registry comprises a unique, large-scale study sample of patients with very recent-onset AF and baseline characteristics relevant in a wide variety of real-world clinical settings.

However, observational studies also have limitations. As patients are non-randomised, confounding is a major challenge. We applied robust statistical methods, including a substantial number of potential confounders in the propensity score weighting scheme. Nevertheless, we cannot rule out residual, unmeasured confounding, as we were not able to account for factors such as social differences, treatment adherence, follow-up resources, and possible local differences in the package of care.

As one of the goals of the study was to observe real-world patterns, all rhythm control strategies chosen by local investigators were allowed and reported as rhythm control. We did not systematically collect data on whether a strategy initiated at baseline changed during follow-up, and thus were unable to analyse treatment cross-over. Moreover, information on the type of AF at the last visit was unavailable for more than one-third of the patients. Success of cardioversion and time in sinus rhythm were not registered. As patients in this study were newly diagnosed and initiated on rhythm control early after diagnosis, the results might not be generalizable to patients who are initiated on rhythm control at a later stage or with long-standing AF. Also, a longer follow-up could have provided important information on long term effects.

## 5 Conclusion

In this large, multinational registry, a rhythm control strategy was initiated at baseline in about half of the patients with newly diagnosed non-valvular AF. A significantly lower risk of all-cause mortality and non-haemorrhagic stroke/SE were observed for patients who received early rhythm control.

## Funding

This work was supported by the Thrombosis Research Institute (London, UK).

## Declaration of Competing Interest

The authors declare the following financial interests/personal relationships which may be considered as potential competing interests: [The authors declare the following potential conflicts of interest: MPK is a recipient of a grant to the institution from Bayer, for support of her ASTORIA study (unrelated to the current publication). TSH reports personal fees from AstraZeneca, Bayer, Boehringer Ingelheim, Bristol-Myers Squibb, Imedic, Novartis, MSD, Sanofi, and Pfizer. DA reports personal fees from Bayer, Boehringer-Ingelheim, Bristol Meier Squibb, MSD and Pfizer, and grants to the institution from Medtronic and BMS. PJ has served as a consultant or on an advisory board for Bayer, Boehringer Ingelheim, and Novartis. AJC has received institutional grant funding and personal fees from Bayer, Boehringer Ingelheim, Bristol Meier Squibb, Daiichi Sankyo, and Pfizer, and personal fees from Sanofi, Menarini, Abbot, Boston Scientific and Medtronic. AKK has received grants and personal fees from Bayer AG, and Sanofi S.A., and Anthos Therapeutics. SH has received personal fees from Bayer, BMS, Daiichi Sankyo, Pfizer, and Sanofi. SG was a recipient of quality personal fees from Jansen and Antos, and Phillips, fees from the American Heart Association as an Associate Editor for Circulation, and Steering Committee fees from Duke University. EP declares honoraria for participation in clinical trials by Pfizer, Bristol-Myers Squibb, Boehringer Ingelheim, Sanofi, AstraZeneca, Daiichi Sankyo Pharma Development, GlaxoSmithKline DMPK. She received fees for contributions to advisory boards or oral presentations from Sanofi, Bayer, Lilly, AstraZeneca, Boehringer Ingelheim, Bayer, Pfizer, Bristol-Myers Squibb, Servier, Takeda-NYCOMED, GlaxoSmithKline, MEDICINES, Aspen, and Stada. PA reports research grants to the institution from Novo Nordisk, Sanofi, and Spark Therapeutics. All other authors have reported that they have no relationships relevant to the content of this paper to disclose.].

## Data Availability

Requests for patient level data can be made to Saverio Virdone, head of statistics at the Thrombosis Research Institute (svirdone@tri-london.ac.uk). These requests should include a protocol summary and a summary of the statistical analysis plan. The request will be reviewed by the data sharing committee for approval and next steps will be discussed with the requestor.
